# Environmental control of invasiveness and metastatic dissemination of tumor cells: the role of tumor cell-host cell interactions

**DOI:** 10.1186/1478-811X-8-24

**Published:** 2010-09-07

**Authors:** Lido Calorini, Francesca Bianchini

**Affiliations:** 1 Dipartimento di Patologia e Oncologia Sperimentali, Università degli Studi di Firenze and Istituto Toscano Tumori (ITT), Italy

## Abstract

Recent advances in tumor biology led to the realization that, in order to understand the mechanisms involved in proliferation and invasion of tumor cells, an analysis of the complex interactions that tumor cells establish with host cells of tumor microenvironment is required. The bidirectional interactions between tumor cells and components of tumor microenvironment, in particular endothelial cells, cells of monocyte/macrophage lineage and fibroblasts/myofibroblasts, play a critical role in most of the events that characterize tumor progression and metastasis. Interactions between these "reactive" normal cells and the genetically altered tumor cells, by either cell-to-cell contacts or soluble mediators, control the most aspects of tumor formation and progression. This review addresses some of the experimental evidences documenting that tumor cells may influence host cells of their own microenvironment by triggering changes that facilitate their local as well as distant dissemination. Therefore, it focuses on macrophages and fibroblasts that, upon stimulation by tumor cells, change their state towards a tumor-promoting-like phenotype.

## Cancer as a non-homogeneous mass

Most primary tumors can be treated successfully by surgery alone or in combination with chemotherapy, immunotherapy or radiotherapy. However, the treatment of disseminating tumors, once they have spread to secondary sites, is a much more difficult task. Effective treatment of multiple metastatic lesions by surgery or radiotherapy is usually impossible due to their distribution in vital organs. Tumor cell invasiveness and metastasis occur by a complex series of events in which malignant cells invade host tissues, penetrate into body cavities, lymphatic and/or blood circulatory systems. Subsequently they disseminate to distant sites where they invade into new surrounding tissues and proliferate to form secondary tumors [1-3]. Metastatic diffusion of cancer cells remains the most important clinical problem, and malignancy is represented by the ability of tumor cells to invade adjacent host tissue at the primary site and then to diffuse and colonize secondary organs.

Epidemiological and experimental evidences suggest that a wide variability exists in the metastatic spread of different human malignancies, but even cancers of the same histological type often produce quite diverse disease progression and survival outcomes for individual patients [4]. This suggests that even in the same histological class of cancer, the expected incidence and localization of metastatic lesions is not completely certain [4,5].

The capacity of cancers to evolve and change during their development has been termed "tumor progression" by L. Foulds (1954) [6]. The biological characteristics that define tumor progression have been extensively described, although the underlying mechanisms are still not completely defined. Tumor cells, during their sometimes decade-long development, accumulate increasingly genetic alterations, which are typically generated by random mutational events, finally allowing them to assume all the characteristics of an invasive and metastatic cancer. In concert with this "genetic instability", a key role in favouring genetic/epigenetic changes in tumor cells is played by local as well as systemic host factors [7-9]. It has been demonstrated that metastatic dissemination can be influenced by diet [10-13], the neuro-endocrine state [14] and inflammatory-reparative processes [15-17]. Among the local factors, particular attention has been devoted to the interactions that tumor cells establish with various noncancerous types of cells that reside in or are attracted to the tumor microenvironment. In particular, the interaction of tumor cells with platelets [18], lymphocytes [19], polymorphonuclear cells [20,21], fibroblasts [22-25] and monocytes/macrophages was proved to be relevant to tumor progression [26,27]. Under certain conditions, stromal cells may inhibit tumor growth, but in other cases, they can stimulate the growth and invasiveness of tumor cells. Therefore, host cells, mainly in proximity to tumor cells, may markedly alter tumor growth and invasiveness. A bidirectional interaction between tumor cells and host cells, is now recognized as crucial for the decision of whether tumor cells progress toward metastatic dissemination or whether they remain dormant [28,29]. Numerous bioactive agents such as proteins of the extracellular matrix, growth factors, cytokines, chemokines and other molecules secreted by host cells contribute to the evolution of tumor cells, including the generation of a metastatic phenotype. It is hence important to recognize that tumors, like normal tissues, are dependent on the formation of a reactive stroma. Nicolson has postulated that the acquisition of a malignant phenotype in tumor cells may be related in part to a phenotypic switch promoted by the host environment and related to quantitative transcriptional or translational changes, resulting in a transient alteration in the concentrations of biologically active products [30]. Thus, both irreversible genetic alterations and transient phenotypic properties contribute to the generation of a malignant phenotype.

Although additional interactions will almost certainly be discovered and the significance of these interactions will be elucidated, it is already at present well accepted that the reactive stroma of cancers is usually associated with increased numbers of fibroblasts, enhanced capillary density and the deposition of a new extracellular matrix that is rich in type-1-collagen and fibrin. Circulating monocytes are also recruited to the reactive stroma in response to the tumoral chemotactic factors and the wound healing-like processes occuring during tumor growth. Tumor-associated macrophages derived from differentiated monocytes and resident macrophages represent the major component of host leukocytes that infiltrate tumor tissues.

This review focuses on the cancer progression towards invasiveness and metastatic spread, taking into consideration the biological role expressed by the so-called "tumor-associated macrophages" (TAM) and "cancer-associated fibroblasts" (CAF).

## Tumor cell - macrophage interactions

The monocyte/macrophage lineage constitutes a large portion of tumor infiltrating host cells. They enter into the tumor mass via blood vessels throughout the life span of tumors, from early-stage nodules just beginning to vascularise to late-stage tumors that are invasive and metastatic [31,32]. A number of tumoral chemoattractants ensure this recruitment, including colony-stimulating factor-1 (CSF-1, also known as M-CSF) [33,34], CC chemokines [35] and vascular endothelial growth factor (VEGF) [36].

Two major lines of evidence connect macrophages and cancer: Firstly the association of chronic inflammation, that leads to macrophage accumulation, with cancer initiation and promotion [15-17]; secondly a high density of TAM correlates with poor prognosis in over 80% of studies [37]. TAM accumulate in critical areas of tumors, such as the hypoxic areas, and hypoxia triggers a pro-angiogenic program in these cells [38]. Hypoxia characterizes the microenvironment of many solid tumors and it has been shown to affect many biological properties of host cells as well as tumor cells that are implicated in tumor growth and metastatic dissemination, e.g. the switch from oxidative to glycolytic metabolism, the production of vascular endothelial growth factors and protease activities [39,40].

Macrophages are remarkable for the diverse activities in which they can engage on different occasions. Many of these activities appear to be opposing each other: pro-inflammatory *vs* anti-inflammatory, immunogenic *vs* tolerogenic, and tissue destructive *vs* tissue reparative processes. In particular, we know that macrophages from healthy or inflamed tissues are capable of lysing tumor cells, presenting tumor-associated antigens to T-cells and expressing stimulatory cytokines for T- and NK-cells [41]. On the other hand, macrophages isolated from experimental as well as spontaneous tumors show a reduced level of cytotoxic activities [26,27]. Clearly, macrophages are multifunctional cells that "adapt" themselves to the stimuli that prevail at the site to which they have been attracted [42]. Quiescent macrophages of tissues (resident macrophages) respond to immune or bacterial stimuli by expressing new functional activities, resulting in their capacity to recognize and destroy transformed cells (activated macrophages). During this transition, macrophages may express a number of discrete phenotypic changes characterized by specific functional activities. It is possible, that the contrasting effects exerted by TAM on the growth and metastatic diffusion of tumor cells may reflect different states of activation acquired by macrophages in the tumoral microenvironment. The plasticity of macrophages may be exploited by tumor cells to elicit distinct functions at different stages of tumor progression. It is also possible that multiple subpopulations of TAM exist within a tumor mass, and these may change during tumor development and on the basis of their location.

**Figure 1:**
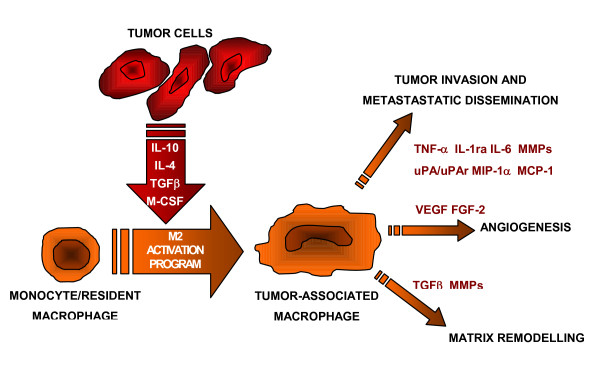
**Some biological activities of polarized M2 macrophages**.

Mantovani *et al*. propose that TAM switch into polarized type II or M2 macrophages [43]. These cells suppress T-cell activity and have poor antigen-presenting capacity, promote proliferation through arginase, angiogenesis and tissue repair (Figure 1), in contrast to classically activated type I or M1 macrophages that are able to kill microorganisms and tumor cells [43]. The signals that lead to the M2 polarization of TAM are not yet completely understood, but IL-10 and TGFβ might play a role [44]. However, a transcriptome analysis of TAM from a mouse fibrosarcoma showed an expression profile of genes that appears to be mostly M2, but with some M1 traits [45]. Sica and Bronte (2007) suggest that a switch from the M1 to the M2 phenotype in TAM might parallel the different interactions that take place between tumor cells and macrophages during tumor progression [46].

The contrasting effects of TAM are well exemplified by the macrophage L-arginine metabolism. Macrophages utilize L-arginine to synthesize nitric oxide (NO) with the help of inducible NO synthase (iNOS), and to produce L-ornithine through arginase activity. While NO may contribute to macrophage-driven tumoricidal activities, the polyamines derived from L-ornithine are essential nutrients for tumor cell proliferation [47]. Thus, the balance between iNOS and arginase activity in TAMs might be critical for tumor progression [48,49]. In addition, arginase can down-regulate NO production by decreasing the intracellular arginine concentration, and low concentrations of NO may act as part of a signalling cascade for neovascularisation [50]. Thus, it was suggested that NO can have pro- or anti-tumor actions, depending on the local concentration of the molecule.

A tumor mass cannot grow beyond 2-3 mm^3 ^in size without angiogenesis. Neovascularization provides an increased supply of nutrients and oxygen, and facilitates the dissemination of tumor cells to distant organs. Most solid tumors pass through two phases of growth: the avascular phase and the vascular phase, when new capillaries penetrate the tumor and it begins to massively grow and invade. Using transgenic mice susceptible to mammary cancer, Lin *et al*. (2006) demonstrated that a reduction of macrophage infiltration delayed the angiogenic switch and the malignant transition of tumor cells [51]. On the other hand, overexpression of CSF-1 in wild-type mice that leads to an early induction of macrophage infiltration into premalignant lesions accompanied by neoangiogenesis, accelerates their transition to malignancy [52]. Vascular endothelial growth factor (VEGF), a key player in the angiogenesis process, is expressed by both tumor cells and TAM in several histological types of human tumors [53]. In addition, TAM-derived inflammatory cytokines (IL-1β, TNFα) may stimulate tumor cells to enhance the production of VEGF [54], and to produce angiogenin, a potent proangiogenic protein [55]. VEGF may become also available in the tumor microenvironment through the release of matrix metalloprotease-9 (MMP-9) by TAM [56].

Recently, Giordano *et al*. demonstrated that TAM are the most important cell type producing semaphorin 4 D within tumor stroma, a molecule required for angiogenesis and vessel maturation [57]. Therefore, TAM are critical in tumor angiogenesis, an essential step in tumor progression and metastatic dissemination [58].

Moreover, using a intravital multiphoton microscopy, Wyckoff *et al*. (2007) observed that tumor cell intravasation in mammary tumors occurs in association with perivascular macrophages [59], and Ojalvo *et al*. (2010) demonstrated that this subset of the macrophage population is particularly enriched for molecules involved in Wnt signaling [60].

Furthermore, tumor cells co-cultivated with macrophages display a higher invasiveness through a TNFα-dependent MMP induction in macrophages [61]. Direct evidence for the role of MMPs in tumor invasiveness has been provided by many studies and cancer cells might stimulate TAM to produce MMPs in a paracrine manner through the secretion of various stimuli, such as interleukins, growth factors and an extracellular matrix metalloproteinase inducer (CD147). The invasiveness of tumor cells is also stimulated by epidermal growth factor (EGF) synthesized by TAM in response to tumor-derived CSF-1, leading to the induction of several genes involved in the migration of tumor cells [62].

The leading edge of a tumor mass is the site where TAM direct the invasion of tumor cells into host tissues. With melanoma cells, we found that the areas of greatest macrophage density were peritumoral [63,64], and using a suitable* in vitro* model we demonstrated that upon contact with melanoma cells, inflammatory macrophages express increased levels of COX-2 [64], uPAR and MMP-9 [65]. It is also possible that MMPs secreted by TAM can be recruited to cancer cell membranes, and are then used as tools by the tumor cells to progress through a specific site [66]. Hiratsuka *et al*. (2002) proved that MMP-9 expressed in alveolar endothelial cells and macrophages renders the pulmonary metastatic site fertile for secondary growth of malignant cells, a mechanism dependent upon the activation of the VEGF-VEGFR signaling cascade [67]. TAM also produce, under the influence of tumor cells, the urokinase plasminongen activator (uPA) and receptor (uPAR), that may cause degradation of ECM to promote invasion and spread of tumor cells [68,69]. The uPA/uPAR system does not only support the invasion of tumor cells, it also modulates cell adhesion by interactions of uPAR with vitronectin and integrins. Therefore, the uPA/uPAR system is endowed with the structural and functional properties required to promote most important mechanisms of tumor cell migration [68,69].

Genetic experiments provide a causal link between CSF-1-dependent TAM and malignancy in mammary and lung cancer [70]. In particular, crossing transgenic mice susceptible to mammary cancer and mice containing a recessive *null *mutation in the CSF-1 gene, Lin *et al*. demonstrated that TAM are necessary for distant organ colonization, the final step of metastatic dissemination [71].

**Figure 2:**
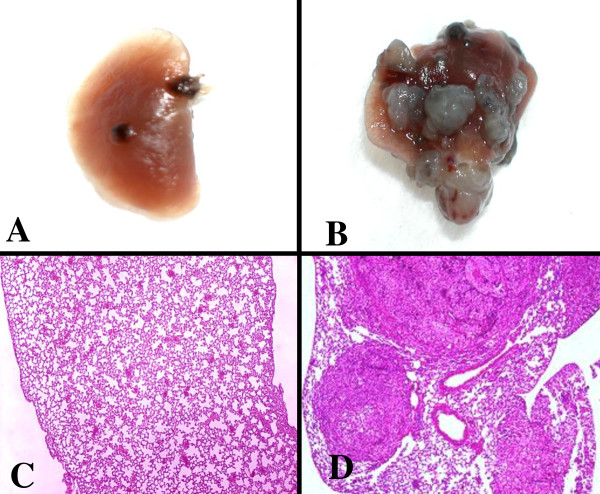
**Change in lung-colonizing potential of B16 murine melanoma cells upon stimulation by TAM**. Surface colonies (A, B) and H&E-stained sections (C, D) of lungs collected from syngeneic animals injected with un-stimulated melanoma cells (A, C) or tumor cells stimulated by macrophages (B, D). See the high number of metastatic lesions in lungs from animal injected with macrophage-promoted tumor cells.

In our laboratory, co-cultivation of tumor cells with 'resident' macrophages, responsive macrophages obtained by the use of thioglycolate broth, or with 'elicited' macrophages obtained by the use of specific infectious agents (*Corynebacterium parvum*, BCG, *Listeria monocytogenes*) enabled us to find that the number of lung colonies detected in mice intravenously injected with melanoma cells were greatly enhanced by co-cultivation of tumor cells with elicited, non-cytotoxic, macrophages prior to injection (Figure 2) [72]. Among the biological properties relevant to the metastatic diffusion, tumor cells exposed to the macrophage-prometastatic activity expressed increased invasiveness, an enhanced capacity to adhere to endothelial cells and an elevated ability to escape NK cells by increasing the expression of MHC class I antigens [73]. This metastatic ability of tumor cells as well as their increased invasiveness, adhesion and MHC expression was found to be transient. Inflammatory cytokines, such as TNFα, contribute to the pro-metastatic activity released by elicited macrophages into their growth media [74].

Important recent studies suggest that macrophages are recruited into the target organs and facilitate metastatic cell seeding. Moreover, blocking macrophage lodgement at the metastatic site limits the growth of metastatic cells, even if metastatic lesions have readily been established [75].

Thus, TAM are powerful tumor promoters, capable to stimulate angiogenesis, invasiveness and subsequent metastatic growth, but also able to set up the sites for metastatic cell seeding.

## Tumor cell - fibroblast interactions

**Figure 3:**
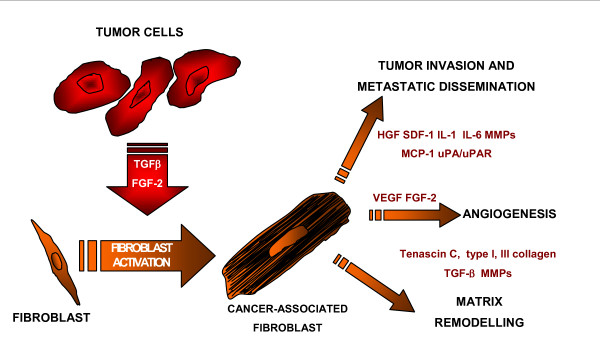
**Some biological activities of CAF**.

Interactions between epithelial cells and stromal cells are crucial in several aspects of normal development, such as growth, differentiation and morphogenesis, but also in pathological conditions, including tumorigenesis. A desmoplastic or stromal reaction characterizes many invasive carcinomas, for example those of the breast, prostate, colon, lung and uterus, and several reports suggest a poorer prognosis associated with carcinomas bearing desmoplastic stroma [76,77]. In 1986, Dvorak described the remarkable similarities between the reactive tumor stroma and the granulation tissue present in areas of inflammation and in tissue undergoing the remodelling phase of wound healing [78]. It was suggested that granulation tissue stimulates tumor cell invasion. Dingemans *et al*. tested this hypothesis and found that a granulation tissue microenvironment, but not normal subcutaneous stroma, elicited an invasive phenotype in tumor cells [79]. The cascade of events leading to a granulation tissue is mainly supported by host fibroblasts, and fibroblasts associated with wound healing as well as reactive tumor stroma (so-called cancer-associated fibroblasts, CAF) are commonly identified by the expression of α-smooth muscle actin (α-SMA). This type of cells was referred to by Gabbiani *et al*. as "myofibroblasts" [80]. Such myofibroblasts, sometimes also termed "activated fibroblasts", participate at all stages of tumor progression. The agents that mediate the transition to myofibroblasts are not yet fully elucidated. In cell culture, myofibroblasts can be induced by transforming growth factor-β (TGFβ), either secreted by tumor cells or host inflammatory cells [77,80]. Proteins of the extracellular matrix produced by stromal cells with myofibroblastic differentiation could act as a barrier against immune cells and may regulate tumor cell behaviour by facilitating cell contacts, motility or transport of nutrients. Moreover myofibroblasts secrete proteins which may stimulate tumor cell invasiveness, angiogenesis and tissue remodelling [81] (Figure 3). Tumor growth and metastasis is significantly reduced in fibroblast-deficient mice, and injection of wild-type fibroblasts into these mice partially reversed the observed phenotype, providing further evidence for the involvement of fibroblasts in the emergence of metastasis.

Regarding secreted factors that affect tumor cell invasiveness, myofibroblasts are a source of extracellular matrix-degrading proteases such as the MMPs. In particular, MMP-3, also known as stromelysin 1, is highly expressed in fibroblasts/myofibroblasts and participates in the cleavage of E-cadherin, thereby prompting epithelial cancer cells to perform an epithelial-mesenchymal transition (EMT) [82]. Cancer cells undergoing EMT loose cell-cell contacts, acquire mesenchymal properties and develop invasive and migratory abilities. Consequently, EMT of cancer cells is recognized as an important determinant of tumor progression [83].

By injecting tumor cell/fibroblast cell suspension into immunodeficent mice, Orimo *et al*. found that CAF isolated from a human breast carcinoma and expressing a myofibroblast phenotype, promote the growth of carcinoma cells through a stromal cell-derived factor-1 [SDF-1]/CXCR4-dependent mechanism. These CAF did not show aneuploidy or *in vivo *tumorigenic activity [84]. Moreover, mammary carcinoma-associated fibroblasts stimulate a high vasculature by recruiting endothelial progenitor cells in tumor xenografts. Thus, SDF-1 secreted by mammary myofibroblasts may stimulate the growth of CXCR4-expressing carcinoma cells as well as angiogenesis [84]. A genome analysis of the stroma of an elevated number of invasive breast carcinoma indicates that the hot spots for mutations in the stroma are not the same as those identified in the epithelium [85]. Therefore, it is possible that an independent pathway of mutation of gene expression works in stromal cells. Recently, Studebaker *et al*. showed that IL-6 secreted by CAF enhances the growth and invasiveness of estrogen receptor α-positive breast carcinoma cells through its effectors, Notch-3, Jagged-1 and carbonic anhydrase IX [86].

Moreover, using an in vitro model of skin carcinogenesis, Cat *et al*. [87] demonstrated that tumor cell-derived TGF-β stimulates reactive oxygen species-dependent expression of α-SMA in skin fibroblasts, and their differentiation into myofibroblasts. This was associated with an increased release of hepatocyte growth factor (HGF), VEGF and IL-6. In view of the notion that skin fibroblasts possess a reduced capacity to secrete IL-6, while senescent fibroblasts strongly up-regulate IL-6 and stimulate malignancy in epithelial cells [88], it was suggested that CAF may represent a subset of senescent fibroblasts. Importantly, a very recent report demonstrates that CAF isolated from dysplastic skin and skin carcinoma express a NF-κB-dependent proinflammatory gene signature responsible for macrophage recruitment, neovascularisation, cancer cell proliferation and invasion. This was also manifest in CAF of mouse and human mammary and pancreatic tumors [89].

**Figure 4:**
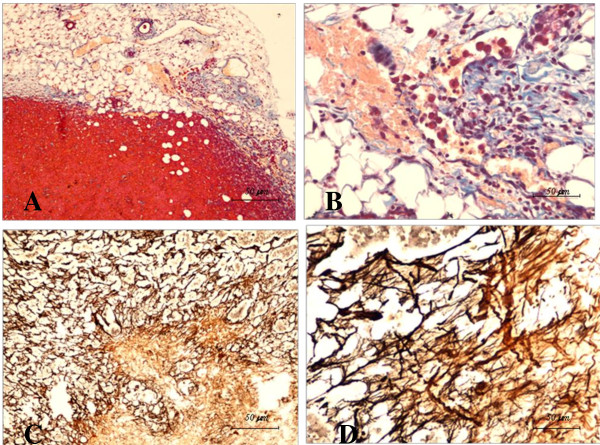
**Stromal organization of a subcutaneous tumor obtained by the co-injection of prostate carcinoma cells (PC3 cells) and "prostate-activated fibroblasts"**. A desmoplastic response surrounding tumor cells rich in a neovasculature (A); and some tumor cells in a large capillary enclosed in a dense collagenous stroma infiltrated by inflammatory cells (B) (Mallory's trichrome). Dense collagen fibers and fine reticular collagen bundles inside the tumor mass (C) and a high magnification of reticulin fibers within the tumor mass (D) (Gomori's method). (Bar 50 μm).

These mesenchymal-epithelial instructive interactions are also responsible for the integrity of the prostate gland. Now, we know that alterations in the complex relationship between prostate epithelial cells and stromal cells contribute to the genomic instability that may promote the progression to a malignant state of these epithelial cells [90]. Some evidence indicates that normal stromal fibroblasts from the fetal urogenital sinus inhibits the *in vivo *growth of prostate tumor cells, when both cell types are inoculated together. In contrast cancer-associated stromal cells co-inoculated with prostate cancer cells promote tumor growth *in vivo *[91]. In Figure 4, tissue sections of reactive stroma around and within a tumor mass are shown that were obtained by the co-injection of prostate adenocarcinoma cells (PC3 cells) and prostate adenocarcinoma-associated fibroblasts into immunodeficient mice. Tumor cells are surrounded by a collagenous stroma particularly enriched in inflammatory cells and new microvessels (A), while collagen fibrils within the tumor are characteristically oriented, in order to sustain the local growth of tumor cells (B). Tuxhorn *et al*. have provided some additional evidence that prostate cancer epithelium stimulates CAF to express vimentin, α-smooth muscle actin and calponin, which is characteristic of the myofibroblast phenotype [92]. Interestingly, initiated non-tumorigenic prostate epithelial cells co-implanted with CAF formed tumors in immunodeficient mice, while CAF do not affect growth of normal human prostate epithelial cells [93].

It is possible that the most important feature in progression of prostate tumors is the ability of tumor cells to stimulate stromal cells to release biological agents for their growth and dissemination. Mutual interactions between carcinoma cells and CAF were reported by Nakamura *et al*.: tumoral IL-1, basic fibroblast growth factor (bFGF) and platelet-derived growth factor (PDGF) stimulate HGF expression in CAF, and in turn, stromal HGF leads to an invasive phenotype in carcinoma cells [94]. Hill *et al*., using a mouse model of prostate carcinoma, showed that tumor cells upregulate p53 in stromal fibroblasts, a process found to induce a selection of a subpopulation of p53 *null *fibroblasts. In turn, selection of a p53 *null *subpopulation of stromal fibroblasts contributed to the progression of carcinoma cells [95].

The origins of CAF were revised by Orimo and Weinberg, who suggested three major alternative hypotheses: a) genetic alteration, b) an activation without genetic alteration of normal tissue fibroblasts, and c) the activation of bone marrow-derived mesenchymal stem cells (MSC) [96]. MSC infiltrate wounds and tumors in high numbers, and, when co-injected into immunodeficient hosts together with weakly metastatic human breast carcinoma cells, they induced in these cancer cells an increase in metastatic potential by a CCL5/CCR5-dependent mechanism [97].

Despite these initial findings, additional efforts at determining the molecular mechanisms that lead to the appearance of differentiated fibroblasts and their multiple contributions in tumor progression are still urgently required.

## Conclusions

Tumor stroma is a specialized form of tissue composed of host cells and signals of different origin, which is associated with tumor cell growth, primarily of epithelial origin. Tumor stroma possesses unique structural features that differ from the native stroma. It is also characterised by a great degree of tumor dependency ("*There is no tumor stroma without a tumor*") and displays a substantial degree of plasticity, with the specific outcomes controlled by tumor cells themselves. Indeed, tumor cells do not only display heterogeneity and induce the expression of signaling molecules that favour their survival and invasiveness into local and distant host tissues, but also influence host stromal elements to produce relevant effectors that act as tumor promoters. Moreover, tumor cell-derived signals recruit and activate some host cells, among which monocytes/macrophages and fibroblasts are the most abundant population within the tumor microenvironment. As was discussed above, both types of cells, macrophages and fibroblasts, are involved in a intricate liaison with tumor cells, that usually leads to tumor progression and activation of the metastatic cascade. Thus, the investigation of the mechanisms that allow macrophages and fibroblasts to contribute to tumor progression, could lead to new approaches for the anti-cancer therapies that are urgently required.

## Competing interests

The authors declare that they have no competing interests.

## Authors' contributions

Both authors contributed to the writing, the conceptual design and the preparation of the figures of this review, and they have read and approved the final manuscript.
